# Acute Complications of COVID-19 With Lasting Damages: A Case of Severe Long-Term Sequelae in a Middle-Aged Female Post COVID-19

**DOI:** 10.7759/cureus.24694

**Published:** 2022-05-03

**Authors:** Mir Sulayman Khan, Srijesa Khasnabish, Nathaniel Grosack, Kevin Mathew, Monissa Rajasri, Roger Stern, Md Y Mamoon

**Affiliations:** 1 Internal Medicine, St. George's University School of Medicine, St. George, GRD; 2 Internal Medicine, New York Institute of Technology College of Osteopathic Medicine, Old Westbury, USA; 3 Internal Medicine, Queens Hospital Center, Queens, USA

**Keywords:** pulmonary embolism (pe), tracheal stenosis, venous thromboembolsim, intubation complication, covid-19

## Abstract

Following coronavirus disease-2019 (COVID-19), many patients experience acute complications and long-term sequelae. Acute complications include respiratory failure, myocardial injury, and neurological complications. Respiratory and thromboembolic complications prove to be acute changes that cause detrimental long-term outcomes. A continued exploration of the COVID-19 hospital course will allow for effective management and treatment of the virus. We report the case of a 48-year-old Hispanic woman who experienced a pulmonary embolism, deep vein thrombosis in all four extremities, and a brain embolus following a COVID-19 infection in 2021. Despite hospital care and prompt treatment, she developed long-term sequelae, specifically post-intubation tracheal stenosis. The critical factor promoting this inflammatory state is the overproduction of cytokines in what is coined a “cytokine storm.” The lasting complications have multiple facets that need to be explored beyond the virus itself. Treatment modalities have their own risks and side effects. Comparing effective and ineffective treatment outcomes for this patient may lead to improvements in COVID-19 management. For this reason, exploring the treatment and complications in the acute setting is necessary for the prevention of the long-term sequelae accompanying cases of COVID-19. While literature exists detailing the unique thrombotic and respiratory complications that can present as a result of COVID-19 coagulopathies, this field is continuously evolving and warrants further research.

## Introduction

The novel coronavirus infection in 2019 (COVID-19) found healthcare workers unprepared to deal with an illness that can cause an array of complications, particularly frequent thromboembolic complications. Severe presentations of COVID-19 resulted in coagulopathies such as pulmonary embolism (PE), deep vein thrombosis (DVT), myocardial infarction, arterial thrombosis, and disseminated intravascular coagulation [1]. Thromboembolic disease increases the risk of mortality. Patients who suffered thromboembolic events during their acute COVID-19 illness had a 2.39 times greater odds of mortality than those who did not [2]. A prompt examination into the care and long-term management of coagulopathies in COVID-19 is necessary to reduce related mortalities. We present a severe case of COVID-19 in a 48-year-old Hispanic woman suffering acute complications of PE, DVTs in all four extremities, and a brain embolus. Her acute issues are followed by a series of complications that resulted in her presentation to our team, specifically tracheal stenosis. Examining the positive and negative aspects of her acute care will allow future providers to discern appropriate treatment modalities that reduce the risk of long-term sequelae and mortality in COVID-19 patients. 

## Case presentation

**Figure 1:**
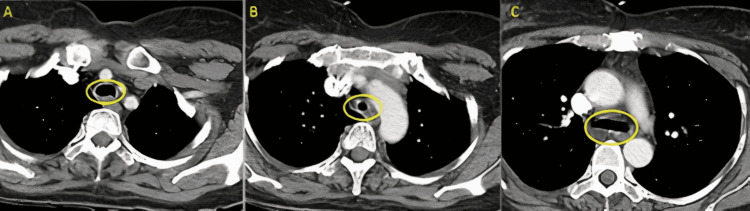
CT Angiogram Pulmonary Embolism with Omnipaque Impression: There is abnormal narrowing and associated abnormal thickening of the wall of the distal trachea. The differential diagnosis includes both inflammatory and neoplastic etiologies. Pulmonary consultation and bronchoscopy is recommended for further evaluation of the trachea. A) Axial view, superior to the level of tracheal stenosis; B) Axial view, at the level of the stenosis; C) Axial view, at the level of the carina and below the level of tracheal stenosis.

**Figure 2:**
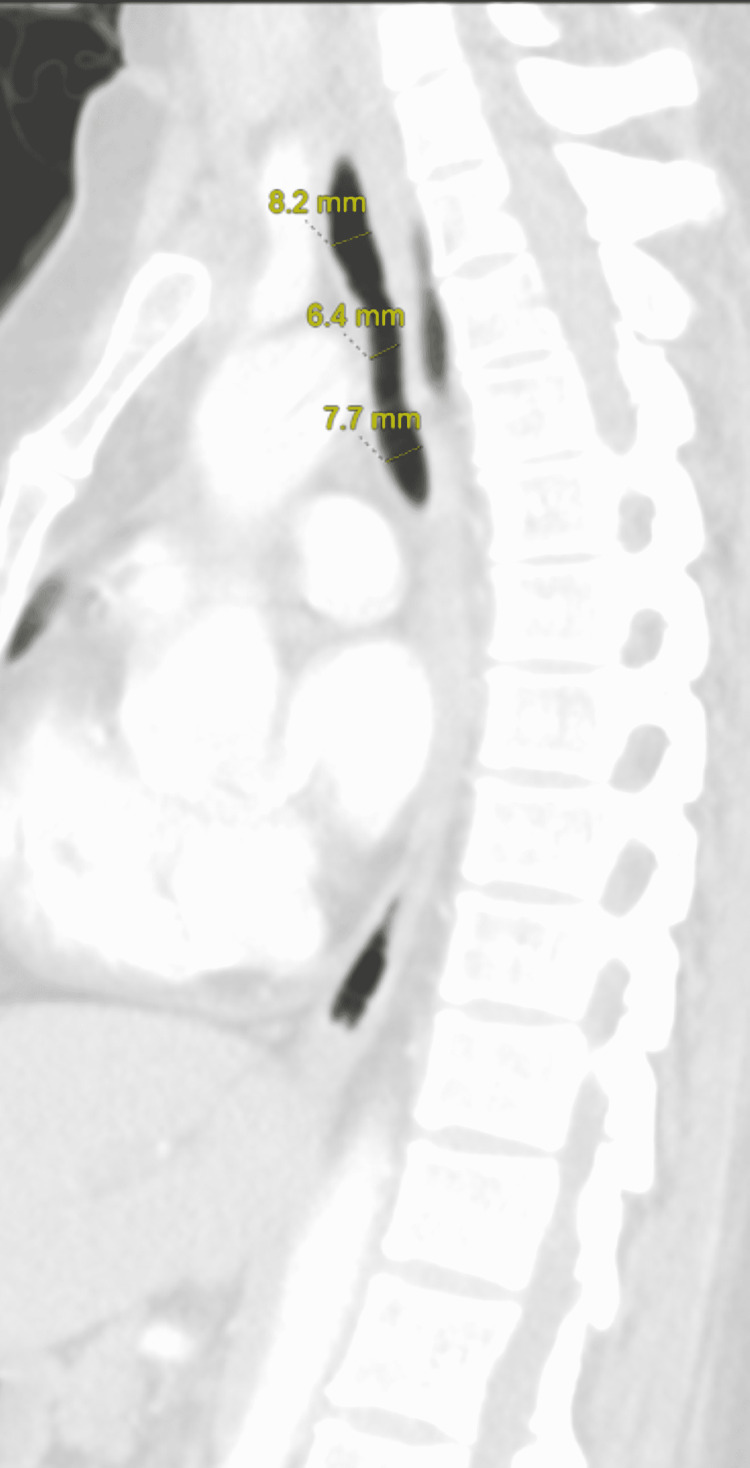
CT Angiogram Pulmonary Embolism with Omnipaque Sagittal view showing distal tracheal stenosis.

A 48-year-old Hispanic female with a past medical history of COVID-19 requiring intubation and tracheostomy presented to the hospital emergency department (ED) complaining of chest tightness. In December 2021, the patient suffered from COVID-19 and was admitted to a hospital in the Dominican Republic (DR). After admission, she received treatment for a PE, DVT in all four extremities requiring thrombectomies, and brain embolus. In January 2022, the patient was intubated for about two weeks and subsequently, a tracheostomy was performed. The patient was discharged on rivaroxaban in late February 2022. Approximately two weeks after discharge, the patient suffered from worsening chest tightness and cough that brought her to the ED. The associated cough was non-productive and present since extubation approximately one month prior. On admission, vitals were as follows: temperature 97.8 degrees Fahrenheit, pulse 92 beats per minute, respiratory rate 18 breaths per minute, blood pressure 125/75 mmHg, and SpO2 97% on room air. On physical exam, mild wheezing was auscultated in the base of the left lung and inspiratory stridor was present in the area of the tracheostomy. Examination of the extremities showed bilateral calf swelling with no pitting edema. Of note, the patient was non-ambulatory secondary to her leg habitus and deconditioning. CT pulmonary angiogram (Figure 1 and Figure 2) revealed the following: 1) an abnormal narrowing and thickening of the wall of the distal trachea and 2) a 6.5 mm nodule within the right middle lobe. See Table 1 for lab values on hospital admission and discharge. The patient provided signed, informed consent for this case discussion. 

**Table 1:** Pertinent Labs Pertinent lab findings on the day of the patient’s admission and day of the patient's discharge.

Parameter	Labs from Admission (March 2022)	Labs from Discharge (March 2022)	Units	Reference Range
White Blood Cell	6.98	8.76	Units/mcL	4.8-10.8
Red Blood Cell	3.71	4.13	Units/mcL	4.2-5.4
Hemoglobin	10.40	11.70	g/dL	12-16
Hematocrit	33.10	37.20	%	37-47%
Mean Corpuscular Volume	89.20	90.10	fL	80-99
Activated Partial Thromboplastin Time	32.10	27.20	Seconds	25.1-36.5
Prothrombin Time	12.40	11.40	Seconds	10-13
International Normalized Ratio	1.00	1.00	--	--
D-Dimer, Fibrinogen Degradation Product	334	--	Ng/mL	<=230

## Discussion

Our patient suffered several long-term sequelae because of a post-inflammatory state secondary to COVID-19 infection and its associated treatments. COVID-19 rapidly activates Th1 cells thereby secreting pro-inflammatory cytokines, particularly interleukin-6 (IL-6) and tumor necrosis factor-alpha (TNF-alpha) [3]. This creates a state of hypercoagulability, promoting detrimental tissue remodeling and clots, in a patient with a clotting risk elevated from baseline. Of note, our patient experienced persistent bilateral leg swelling in the setting of thrombectomies for DVT and tracheal narrowing. Additional findings included the chronic cough and incidental pulmonary nodule finding that was not present on previous imaging from the DR. Understanding the chronic complications that this patient suffered required research into the acute care that this patient received in the DR, which was acquired via contacting the patient’s family and hospital that cared for her initially.

First, her case warranted intubation and subsequent tracheostomy. The benefits of tracheostomy include reducing the length of time for patients requiring mechanical ventilation and intensive care unit level of care [4]. The question remains as to whether the tracheal stenosis was secondary to prolonged intubation. Studies show that post-intubation tracheal stenosis is a rare complication, occurring in 10-22% of cases [5, 6]. However, the risk of post-intubation laryngotracheal stenosis is not reduced with early tracheostomy [7]. In this circumstance, the patient's lengthy intubation and subsequent tracheostomy may not be culpable in the formation of the tracheal stenosis. In an attempt to resolve this patient’s tracheal stenosis, rigid bronchoscopy with balloon tracheoplasty, steroid injections, and tracheal dilation were performed in late March. Preliminary studies support this minimally invasive approach because it significantly improves airway patency [8]. CT neck with and without contrast after initial bronchoscopy showed no evidence of laryngeal or tracheal stenosis. The imaging was, however, limited and did not capture the distal trachea. Four days after bronchoscopy, the patient’s chest tightness resolved and she complained only of an occasional cough. She was deemed ready for discharge and was sent home on rivaroxaban 20 mg. The patient is scheduled for a follow-up bronchoscopy in six weeks (in mid-April). 

Intubation, tracheostomy, and severe COVID-19 inflammatory changes contributed to the remodeling and narrowing of the trachea. Possible future management considerations that can be made to reduce tracheal stenosis include identification of narrow airways, appropriate endotracheal tube sizing, and careful planning prior to the tracheostomy stage to avoid prolonged intubation [9]. Given that this patient was intubated in the DR, we are uncertain whether her tube size was adequate. 

Furthermore, it is important to consider the benefit of non-invasive ventilation and prone positioning prior to intubating patients battling COVID-19. This includes bilevel positive airway pressure devices, continuous positive airway pressure devices, non-rebreather masks, and high flow nasal cannula, which are associated with improved outcomes for these patients [10].

The patient’s lower extremity deconditioning due to prolonged hospitalization is further compounded by the lack of resolution of DVT following multiple thrombectomies. Early prophylactic anticoagulation is pivotal in preventing the thrombotic complications associated with COVID-19 [11]. It is critical that prothrombotic states are identified early on with careful monitoring of D-Dimers and acute phase reactants, including IL-6, TNF-alpha, fibrinogen, and C-reactive protein. Further measures can be taken to improve the patient’s condition such as compression stockings to reduce post-thrombotic syndrome and early ambulation with the assistance of physical therapy. However, our report is limited by the lack of knowledge regarding when interventions were made during the patient’s care in the DR.

## Conclusions

This 48-year-old patient’s complicated COVID-19 course revealed PE, DVT, and a brain embolus, while long-term she experienced tracheal stenosis and lower extremity deconditioning. Ultimately, the long-term ramifications of COVID-19 and its treatments require careful research. Insight into this patient’s response to specific treatment options will allow future providers to prescribe better treatments with improved outcomes. First, understanding the role of intubation duration, technique, and planning is crucial in preventing long-term sequelae like that of post-intubation stenosis. Simultaneously, timely intervention is necessary to prevent thrombotic complications, as is prophylaxis to prevent DVT recurrence. Beyond medical management, it is necessary to facilitate ambulation via physical therapy following prolonged hospital stays related to COVID-19 infection.
